# Words are just Noise, let your Actions Speak: Impact of Nonverbal Communication on Undergraduate Medical Education

**DOI:** 10.12669/pjms.37.7.4180

**Published:** 2021

**Authors:** Anbreen Aziz, Farah Farhan, Faiza Hassan, Aasma Qaiser

**Affiliations:** 1 Dr. Anbreen Aziz, BDS, MHPE Assistant Professor, Department of Dental Health Professions Education and Research, Army Medical College/ Armed Forces Institute of Dentistry (AFID) National University of Medical Sciences (NUMS), Rawalpindi, Pakistan; 2 Dr. Farah Farhan Assistant Professor, Department of Oral Pathology, Foundation University College of Dentistry, Islamabad, Pakistan; 3 Prof. Dr. Faiza Hassan Department Oral Pathology, Rawal Institute of Health Sciences, Islamabad, Pakistan; 4 Dr. Aasma Qaiser Assistant Professor, Department of Medical Education, Kharian Medical College, Combined Military Hospital, Kharian, Punjab, Pakistan

**Keywords:** Nonverbal communication, Kinesics, Facial expressions, Undergraduate Medical Education, Environment

## Abstract

**Objective:**

To explore student’s perceptions regarding impact of kinesics (facial expressions, gestures, head movements and postures) on undergraduate medical education.

**Methods:**

A qualitative exploratory online survey was conducted from July-Sept 2020 among purposively chosen final year BDS students (n=150) of three dental colleges of Islamabad during COVID-19 lockdown phase. Semi-structured survey questions were validated and piloted before execution. Thematic analysis was performed, and consensus was built among all authors regarding findings, hence ensuring analytical triangulation.

**Results:**

Response rate was 46% (69/150). Twenty sub-themes emerged under three domains of kinesics. Participants told that ‘neutral expressions’ frequently used by teachers create ‘boring learning environment’ and ‘hesitation among students to ask questions.’ A smile of teacher imparts ‘new degree of interest in the subject’ and gives ‘freedom of expression’ to the students. On contrary, anger ‘demotivate’ students, instills ‘fear among them’, make them anxious therefore, they are ‘unable to understand lectures’ which ultimately leads to ‘loss interest in the subject’. Use of gestures by teachers creates ‘enjoyable teaching-learning process’ but movements such as clearing throat or shaky legs produce ‘constant split-second interruption’. Moreover, standing posture of teachers bring ‘interest and alertness among students’.

**Conclusion:**

Nonverbal communication can have positive or negative impact on undergraduate medical education. Therefore, teachers may start lecture with a smile and anger should be avoided to produce friendly and healthy learning environment. Faculty training is required for the effective use of nonverbal communication strategies to create an optimal learning environment for the students.

## INTRODUCTION

Humans are socially connected to each other with the help of communication which is the greatest gift by Allah.^1^,^2^ Communication comprises of verbal and nonverbal types.^2^ Verbal communication remained a fundamental skill to be taught and learned in medical field.^3^ It constitutes 45% proportion of human communication which involves spoken words (38%) and tone of voice (7%).^1^,^2^ Whereas, nonverbal communication (NVC) or body language constitutes 55% proportion of human communication and is attracting medical education community from the past three decades.^4^ NVC is an important skill needed for the teachers while they are interacting to large group of students because it effects positively on student’s mood.^1^

NVC is the behavior of the face, body, or voice^2^,^5^ and is divided into; kinesics (body postures and body movements), vocalics (non-linguistic vocal cues such as volume and sound pitch), *oculesics* (gaze and movement of eye), haptics (body contact such as handshakes) and proxemics (personal space and distance).^4^ The word kinesics refers to the study of hand, arm, body, and face movements.^6^ It is one of the most powerful ways through which humans can communicate nonverbally.^7^

Sufficient literature is available on effective use of NVC in the fields of Islamic studies,^8^ business,^9^ psychology,^10^ patient-doctor interactions^11^ or the student’s interactions with simulated patients. But limited literature is available regarding its use as a teaching strategy by medical educators.^5^ Therefore, our study qualitatively explores the impact of kinesics on undergraduate medical students and learning environment. The purpose of this study was to create awareness among medical educators regarding correct use of NVC strategies. Apart from this, it might be of use to curriculum developers at institutional level to integrate the effective use of NVC as a teaching strategy along with other strategies within the curriculum.

## METHODS

An exploratory research using online survey having semi-structured questions was carried out from July-Sept 2020. Ethical permission was taken from institutional review committee of three private dental colleges of Islamabad (Ref. No. IIDC/IRC/2020/003, dated July 02, 2020 and Ref. IMDC/DS/OG/336, dated July 28, 2020, FF/FUMC/215-1/PHY/20, dated Sept 10, 2020).

### Participants

Final year students being the senior most class were purposively included to get in-depth information and students from other years were excluded. Survey was shared through the institutional WhatsApp group of each institute. Iterative data collection and analysis showed that majority of the participants were quite expressive.

### Questionnaire and data collection

Survey questions were developed on google forms following guidelines of AMEE 87 for questionnaire development.^12^ First step was conducting literature review regarding NVC. Second step was telephonic interviews with students (n=06) regarding their concepts about NVC. Third step was development of ten items under three themes (face expressions, gestures, head movements and postures) related to kinesics. Fourth step was to get the survey validated from four content experts. The items were then re-arranged, re-phrased and omitted as required. Lastly, pilot testing was conducted (n=10) to manage any technical errors and to see comprehension level of students. After minimal corrections, the survey consisting of six items was approved by all authors and was executed along with informed consent within the survey to ensure confidentiality and anonymity. The data were coded by first author and then shared with other authors for data analysis.

### Data analysis

Frequencies and percentages were calculated for demographics while manual thematic analysis was performed for open-ended questions using systematic approach for qualitative text analysis.^13^ It was done by all authors and proceeded in the following steps:

1. Data was prepared by making a separate word document and pasting text replies of all participants (69) against each open-ended question and each participant’s text reply was labelled with participant number to ensure anonymity. Each question was labelled with theme denoting main domains of kinesics e.g., theme-1 denoted impact of facial expressions, theme-2 dealt with impact of gestures and theme-3 was regarding impact of head movements and postures.
2. All the authors familiarized with data by carefully reading each line and segment to develop in-vivo analytic codes (1^st^ coding cycle) under each category.^14^ Later, codes were arranged, and subcategories (sub-themes) were formed. All the data under each category were again coded (2^nd^ coding cycle).
3. Subcategories were reevaluated and consensus was made among all authors to get diverse perspectives and to ensure analytical triangulation.^15^ Results are presented along with quotes from the original participant’s text replies (Table II).

**Table II:** Impact of Kinesics on Undergraduate Medical Education.

*Subthemes*	*Theme-1: Impact of Teacher’s Facial Expressions*
***Neutral Facial Expressions***	
Lack of distraction	‘Neutral expressions do not give us a great deal of information about the teacher’s mood or feelings…They do help to stay focused’ (F, A1, P#27)
Hesitation in asking questions	‘Creates hesitation in us and makes it difficult to ask questions… results in tense environment’ (F, A1, P#43)
Boring learning environment	‘They make the lectures boring and I lose interest’ (F, A2, P#17)
***Smile on Teacher’s Face***	
Freedom of expression	‘Smiling face of teacher creates a positive vibe among students and they become fearless to express everything’ (F, A2, P#10) ‘There is no fear of insult or harsh words from smiling teacher’ (F, A1, P#38)
New degree of interest in the subject	‘A genuine smile adds a new degree of interest in the subject’ (F, A2, P#17)
Building sense of security and respect	‘Smile is very essential for developing a sense of security and healthy environment for students… We literally enjoy those teachers who start lecture with welcoming gestures rather than just starting topic directly… We get more closer to them and build unconscious sense of respect and friendliness towards them’ (F, A1, P#28)
Motivation	‘It really keeps me motivated to listen to every bit of the lecture…teacher put so much conscious effort…I should pay back as well’ (F, A1, P#6)
Barrier-free and pleasant learning environment	‘It makes us feel comfortable and welcomed. It becomes easier to communicate and ask questions’ (F, A1, P#43) ‘Smile is contagious, and it spread happiness among the students and create barrier-free learning environment’ (F, A1, P#69) ‘A genuine smile makes my days of learning much pleasant without any doubt’ (F, A1, P#46)
***Anger on Teacher’s Face***	
Fear among students	‘Anger instills fear for no reason and students are afraid of asking questions’ (M, A2, P#2) ‘Students develop fear of possible insult resulting in reaction to minor mistake’ (F, A2, P#9)
Difficulty in understanding lectures	‘It makes students anxious and, in that anxiety, it is sometimes difficult to understand lectures’ (F, A2, P#12)
Loss of interest in the subject	‘It has negative effect on my learning experience as it results in loss of interest in the subject’ (F, A2, P#17) ‘I have a habit of asking lot of questions and bursting out answers without a second thought. I have to forcefully hold my tongue and refrain from asking questions if a teacher looks in a sour mood… Participation goes down drastically’ (F, A1, P#20)
Demotivation	‘Anger is discouraging and makes learning just a burden for us’ (F, A1, P#39)
Communication barrier and hostile environment	‘Anger will lead to communication gap between tutor and student. It can make the classroom a hostile environment to work in’ (M, A2, P#27)

***Theme-2: Impact of Teacher’s Gestures***	

***Hand Gestures***	
Clarification of concepts	‘They clarify concepts by accompanying speech for a better learning’ (F, A2, P#8)
‘Hand gesture is a form of self-expression. Teachers usually take aid from them to explain a concept better’ (M, A2, P#23)
Enjoyable teaching and learning process	‘Hand and face gestures gives this feeling that teacher is enjoying teaching and somehow making students enjoy the lecture too. It depends how well a teacher calibrates his/ her verbal and nonverbal communication pattern to engage with a class’ (F, A1, P#20)
***Behavior or movements***	
***(clicking pens, shaking legs, self-touching, throat clearing sounds, glancing at watch or clock etc.)***	
Natural human behavior	‘They are just random gestures that makes the teacher look more natural rather than a robot. I would choose a teacher with excessive gestures and expressions over a stiff teacher with zero expressions and gestures’ (F, A1, P#20)
Split second interruption	‘Such movements do affect learning environment. If the teacher keeps looking at clock or is always clearing throat or shaky legs; these add to the negative aspect of the teacher’s personality. If the teacher keeps clearing throat, it means a constant split-second interruption during the lecture and the students will lose focus’ (M, A1, P#25)

***Theme-3: Impact of Teacher’s Head Movements and Postures***	

***Head Movements***	
Confidence	‘I feel appreciated and confident when teacher moves head in favor of my answer’ (F, A2, P#52)
***Standing Posture***	
Imparting interest and alertness	‘It increases interest and alertness among students when teacher is standing and bring energy in students’ (F, A1, P#45)
Less distraction	‘I think standing and pacing around the classroom has the best impact as it stops you from zoning out or getting distracted by anything else’ (F, A2, P#24)
Serious learning environment	‘Standing creates a serious learning environment’ (F, A2, P#34)

*M-Male, F-Female, A-Age group (1-2), P-participant #.

## RESULTS

Sixty-nine students (46%) responded out of 150 final year students from three dental institutes. The respondents were predominantly females (88.4%), with age groups 20-22 years (55%) and 23-25 years (45%) (Table-I).

**Table I:** Characteristics of the study participants (N=69).

*Characteristics*		*Frequency N*	*Percentage (%)*
Gender	Male	8	11.6
	Female	61	88.4
Age Groups (Years)	A1: 20-22	38	55
	A2: 23-25	31	45

Our study has explored the student’s perceptions about the impact of kinesics (facial expressions, gestures, head movements and postures) on undergraduate medical students and learning environment. Twenty subthemes emerged under three main themes after thematic analysis of the data.

## DISCUSSION

The study has explored student’s perceptions regarding impact of kinesics (facial expressions, gestures, head movements and postures) on undergraduate medical education including educational environment. Comparing with a previous study held on teachers at government secondary schools of Peshawar district of Pakistan^16^, most of our findings are same showing that kinesics have impact on students and their learning environment. Common findings are motivating learning environment due to smile on a teacher’s face.^16^ Learning environment impacts attitude, knowledge, skills, and academic achievements of medical students.^17^

In contrast, our study has highlighted other aspects such as difficulty in communication and understanding lecture due to anger on teacher’s face that makes the environment hostile and learning becomes burden for students. Teacher’s respect is undermined by anger and it adversely effects teachers themselves too.^18^ Moreover, it is reported in this study that anger demotivates students and instills fear among them. Whereas on the other hand, smile on teacher’s face reduces fear of being insulted by teachers upon giving wrong answer to a question and keep them motivated to listen to every bit of a lecture. In literature, a study explained beautifully that smile used in a classroom setting was powerful to invite student’s smile^19^ as one of the participants said that teacher’s smile is contagious, and it spreads happiness among students.

Our study has shown that hand gesture to indicate size or shape of an object when accompany speech helps in better learning as given in the previous studies too.^20^,^21^ The participants said that hand and face gestures make them feel that teacher enjoys teaching and somehow making students enjoy the lecture too. Therefore, gestures are center of importance in communication as they bring energy to the speech.^22^ Moreover, one of the participants emphasized that it depends how well a teacher calibrates his/ her verbal and nonverbal communication pattern to engage him/ herself with a class. Among behavior or movements, looking at watch or clock, clearing throat, shaking legs etc. got mixed comments. Few participants were of view that they are just random gestures making a teacher look more natural rather than a robot with zero expressions. While few of the participants said that these movements add to negative aspect of the teacher’s personality. They further added that these movements may cause split second interruption during the lecture and students may lose focus.

Standing posture used by majority of the teachers though create serious learning environment but impart interest and alertness among students and lessens their distraction. Every position or movement of teacher while teaching matters to great extent as it sends message to the students.^23^ Therefore, in order to send correct message, a teacher should be able to use correct position for better teaching and learning experience.^23^

### Strengths of the study

Our study has highlighted positive and negative impact of teacher’s nonverbal communication on medical students and learning environment. Hence, this study is unique as the data were collected from the relevant stakeholders to get in-depth perspectives.

### Limitations of the study

The data could have been collected from the relevant stakeholders from other health professions such as medicine, nursing, pharmacy, and physiotherapy to get broad perspectives.

## CONCLUSION

Nonverbal communication strategies should be used correctly by medical teachers to produce positive impact on learners and learning environment. Teachers should be enthusiastic and in happy mood while communicating with students within a classroom setting. Therefore, teachers may start lecture with a smile and anger should be avoided to produce friendly and healthy learning environment. Gestures create enjoyable teaching-learning process and the standing type of posture gathers student’s attention and creates active learning environment. Faculty training is required for the effective use of nonverbal communication strategies to create an optimal learning environment for the students. Future research could collect data from students of other health professions as well to gain broader perspectives. Moreover, a scale could be developed which can measure satisfaction and happiness of the students regarding nonverbal communication strategies used by their teachers.

### Author’s contribution:

**AA:** Conception of study, literature review, questionnaire development, data analysis and interpretation.

**FF:** Drafting manuscript, pilot testing.

**FH:** Data collection, contribution in write-up,

**AQ:** Literature review, Data analysis.

All authors are responsible and accountable for accuracy or integrity of the research work.
